# *N*-(2,6-Dimethyl­phen­yl)-2-methyl­acetamide

**DOI:** 10.1107/S1600536807068419

**Published:** 2008-01-09

**Authors:** B. Thimme Gowda, Sabine Foro, Hartmut Fuess

**Affiliations:** a Department of Chemistry, Mangalore University, Mangalagangotri 574 199, Mangalore, India; b Institute of Materials Science, Darmstadt University of Technology, Petersenstrasse 23, D-64287 Darmstadt, Germany

## Abstract

The structure of the title compound (26DMPMA), C_11_H_15_NO, is closely related to the side-chain unsubstituted *N*-(2,6-dimethyl­phen­yl)acetamide and side-chain substituted *N*-(2,6-dimethyl­phen­yl)-2,2,2-trimethyl­acetamide and 2-chloro-*N*-(2,6-dimethyl­phen­yl)acetamide, with slightly different bond parameters. The mol­ecules in 26DMPMA are linked into chains through N—H⋯O hydrogen bonding.

## Related literature

For related literature, see: Gowda *et al.* (2004 ▶, 2008 ▶); Gowda, Foro & Fuess (2007 ▶); Gowda, Svoboda & Fuess (2007 ▶).
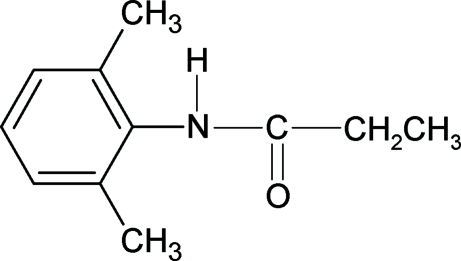


## Experimental

### 

#### Crystal data

C_11_H_15_NO
*M* *_r_* = 177.24
Monoclinic, 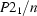

*a* = 4.7915 (7) Å
*b* = 11.593 (2) Å
*c* = 17.966 (3) Å
β = 96.11 (2)°
*V* = 992.3 (3) Å^3^
*Z* = 4
Mo *K*α radiation
μ = 0.08 mm^−1^
*T* = 100 (2) K
0.50 × 0.14 × 0.08 mm

#### Data collection

Oxford Diffraction Xcalibur diffractometer with Sapphire CCD detector
Absorption correction: multi-scan (*CrysAlis RED*; Oxford Diffraction, 2007 ▶)*T*_min_ = 0.951, *T*_max_ = 0.989
7811 measured reflections
2005 independent reflections
1262 reflections with *I* > 2σ(*I*)
*R*_int_ = 0.036

#### Refinement

*R*[*F* ^2^ > 2σ(*F* ^2^)] = 0.033
*wR*(*F* ^2^) = 0.080
*S* = 0.95
2005 reflections
164 parameters
Only H-atom coordinates refined
Δρ_max_ = 0.17 e Å^−3^
Δρ_min_ = −0.16 e Å^−3^

### 

Data collection: *CrysAlis CCD* (Oxford Diffraction, 2007 ▶); cell refinement: *CrysAlis RED* (Oxford Diffraction, 2007 ▶); data reduction: *CrysAlis RED*; program(s) used to solve structure: *SHELXS97* (Sheldrick, 2008 ▶); program(s) used to refine structure: *SHELXL97* (Sheldrick, 2008 ▶); molecular graphics: *PLATON* (Spek, 2003 ▶); software used to prepare material for publication: *SHELXL97*.

## Supplementary Material

Crystal structure: contains datablocks I, global. DOI: 10.1107/S1600536807068419/dn2305sup1.cif

Structure factors: contains datablocks I. DOI: 10.1107/S1600536807068419/dn2305Isup2.hkl

Additional supplementary materials:  crystallographic information; 3D view; checkCIF report

## Figures and Tables

**Table 1:** Hydrogen-bond geometry (Å, °)

*D*—H⋯*A*	*D*—H	H⋯*A*	*D*⋯*A*	*D*—H⋯*A*
N1—H1N⋯O1^i^	0.889 (14)	2.065 (14)	2.9352 (15)	165.9 (12)

Symmetry code: (i) 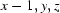.

**Fig. 1.**
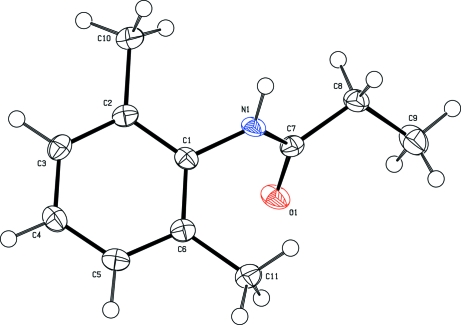
Molecular structure of the title compound showing the atom labelling scheme. Displacement ellipsoids are drawn at the 50% probability level. H atoms are represented as small spheres of arbitrary radii.

**Fig. 2.**
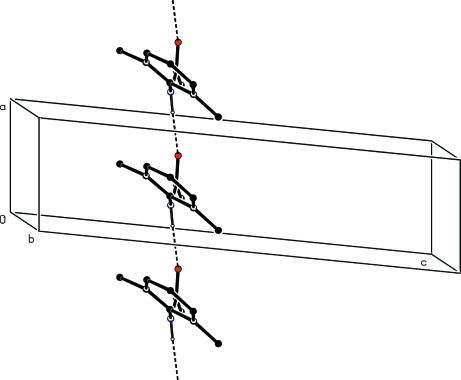
Partial packing view showing the formation of a chain. Hydrogen bonds are represented as dashed lines. H atoms not involved in hydrogen bondings have been omitted for clarity.

## supplementary crystallographic information

### Comment

In the present work, the structure of 2-methyl-*N*-(2,6-dimethylphenyl)- acetamide (26DMPMA) (Fig. 1) has been determined as part of a study of the effect of ring and side chain substitutions on the solid state geometry of biologically significant compounds such as acetanilides (Gowda, Foro & Fuess, 2007); Gowda, Svoboda & Fuess, 2007); Gowda *et al.*, 2008). The structure of 26DMPMA is closely related to the side chain unsubstituted *N*-(2,6-dimethylphenyl)-acetamide (26DMPA) (Gowda, Foro & Fuess, 2007) and side chain substituted, 2,2,2-trimethyl-*N*-(2,6-dimethylphenyl)-acetamide (26DMPTMA) (Gowda, Svoboda & Fuess, 2007) and 2-chloro-*N*-(2,6-dimethylphenyl)- cetamide (26DMPCA) (Gowda *et al.*, 2008). The bond parameters in 26DMPMA are similar to those in 26DMPA, 26DMPTMA, 26DMPCA and other acetanilides (Gowda, Foro & Fuess, 2007; Gowda, Svoboda & Fuess, 2007; Gowda *et al.*, 2008). The molecules in 26DMPMA are linked into infinite chains through N—H···O hydrogen bonding (Table 1 and Fig.2).

### Experimental

The title compound was prepared according to the literature method (Gowda *et al.*, 2004). The purity of the compound was checked by determining its melting point. The compound was further characterized by recording its infrared and NMR spectra (Gowda *et al.*, 2004). Single crystals of the title compound were obtained from a slow evaporation of an ethanolic solution and used for X-ray diffraction studies at room temperature.

### Refinement

The H atoms were located in difference map, and their positional parameters were refined freely with N—H = 0.89 (1) %A and C—H = 0.96 (1)–1.02 (2) Å. All H atoms were refined with isotropic displacement parameters (set to 1.2 times of the *U*_eq_ of the parent atom).

### Crystal data

**Table:**

C_11_H_15_NO	*F*_000_ = 384
*M**_r_* = 177.24	*D*_x_ = 1.186 Mg m^−^^3^
Monoclinic, *P*2_1_/*n*	Mo *K*α radiation λ = 0.71073 Å
Hall symbol: -P 2yn	Cell parameters from 1603 reflections
*a* = 4.7915 (7) Å	θ = 2.1–24.9º
*b* = 11.593 (2) Å	µ = 0.08 mm^−^^1^
*c* = 17.966 (3) Å	*T* = 100 (2) K
β = 96.11 (2)º	Needle, colourless
*V* = 992.3 (3) Å^3^	0.50 × 0.14 × 0.08 mm
*Z* = 4	

### Data collection

**Table:**

Oxford Diffraction Xcalibur diffractometer with Sapphire CCD detector	2005 independent reflections
Radiation source: fine-focus sealed tube	1262 reflections with *I* > 2σ(*I*)
Monochromator: graphite	*R*_int_ = 0.036
*T* = 100(2) K	θ_max_ = 26.4º
Rotation method data acquisition using ω and φ scans	θ_min_ = 2.9º
Absorption correction: multi-scan[CrysAlis RED (Oxford Diffraction, 2007); empirical (using intensity measurements) absorption correction using spherical harmonics implemented in SCALE3 ABSPACK scaling algorithm]	*h* = −5→5
*T*_min_ = 0.951, *T*_max_ = 0.989	*k* = −13→14
7811 measured reflections	*l* = −22→21

### Refinement

**Table:**

Refinement on *F*^2^	Hydrogen site location: difference Fourier map
Least-squares matrix: full	Only H-atom coordinates refined
*R*[*F*^2^ > 2σ(*F*^2^)] = 0.033	*w* = 1/[σ^2^(*F*_o_^2^) + (0.043*P*)^2^] where *P* = (*F*_o_^2^ + 2*F*_c_^2^)/3
*wR*(*F*^2^) = 0.080	(Δ/σ)_max_ = 0.001
*S* = 0.95	Δρ_max_ = 0.17 e Å^−^^3^
2005 reflections	Δρ_min_ = −0.16 e Å^−^^3^
164 parameters	Extinction correction: SHELXL97 (Sheldrick, 1997), Fc^*^=kFc[1+0.001xFc^2^λ^3^/sin(2θ)]^-1/4^
Primary atom site location: structure-invariant direct methods	Extinction coefficient: 0.016 (2)
Secondary atom site location: difference Fourier map	

### Special details

**Table:**

Geometry. All e.s.d.'s (except the e.s.d. in the dihedral angle between two l.s. planes) are estimated using the full covariance matrix. The cell e.s.d.'s are taken into account individually in the estimation of e.s.d.'s in distances, angles and torsion angles; correlations between e.s.d.'s in cell parameters are only used when they are defined by crystal symmetry. An approximate (isotropic) treatment of cell e.s.d.'s is used for estimating e.s.d.'s involving l.s. planes.
Refinement. Refinement of *F*^2^ against ALL reflections. The weighted *R*-factor *wR* and goodness of fit *S* are based on *F*^2^, conventional *R*-factors *R* are based on *F*, with *F* set to zero for negative *F*^2^. The threshold expression of *F*^2^ > σ(*F*^2^) is used only for calculating *R*-factors(gt) *etc*. and is not relevant to the choice of reflections for refinement. *R*-factors based on *F*^2^ are statistically about twice as large as those based on *F*, and *R*- factors based on ALL data will be even larger.

### Fractional atomic coordinates and isotropic or equivalent isotropic displacement parameters (Å^2^)

**Table:**

	*x*	*y*	*z*	*U*_iso_*/*U*_eq_	
O1	0.65780 (18)	0.08126 (8)	0.39277 (5)	0.0262 (3)	
N1	0.2247 (2)	0.15987 (9)	0.36988 (6)	0.0189 (3)	
H1N	0.044 (3)	0.1485 (11)	0.3751 (7)	0.023*	
C1	0.3212 (2)	0.27503 (11)	0.36006 (6)	0.0176 (3)	
C2	0.2508 (3)	0.36018 (11)	0.41017 (7)	0.0190 (3)	
C3	0.3532 (3)	0.47138 (12)	0.40192 (7)	0.0227 (3)	
H3	0.310 (3)	0.5309 (11)	0.4368 (7)	0.027*	
C4	0.5195 (3)	0.49761 (13)	0.34589 (7)	0.0240 (3)	
H4	0.592 (3)	0.5742 (12)	0.3436 (7)	0.029*	
C5	0.5822 (3)	0.41284 (12)	0.29609 (7)	0.0230 (3)	
H5	0.699 (3)	0.4338 (10)	0.2572 (7)	0.028*	
C6	0.4831 (3)	0.30035 (11)	0.30172 (7)	0.0196 (3)	
C7	0.4008 (3)	0.07132 (11)	0.38927 (7)	0.0191 (3)	
C8	0.2643 (3)	−0.04089 (12)	0.40805 (8)	0.0226 (3)	
H8A	0.233 (3)	−0.0348 (11)	0.4619 (8)	0.027*	
H8B	0.078 (3)	−0.0457 (11)	0.3814 (7)	0.027*	
C9	0.4399 (3)	−0.14537 (14)	0.39496 (10)	0.0340 (4)	
H9A	0.633 (4)	−0.1371 (13)	0.4197 (8)	0.051*	
H9B	0.362 (3)	−0.2172 (13)	0.4137 (8)	0.051*	
H9C	0.457 (3)	−0.1568 (12)	0.3394 (9)	0.051*	
C10	0.0701 (3)	0.33281 (14)	0.47103 (8)	0.0247 (4)	
H10A	−0.132 (3)	0.3315 (11)	0.4530 (8)	0.037*	
H10B	0.109 (3)	0.2566 (14)	0.4935 (7)	0.037*	
H10C	0.096 (3)	0.3906 (12)	0.5110 (8)	0.037*	
C11	0.5511 (3)	0.21070 (14)	0.24562 (8)	0.0257 (4)	
H11A	0.408 (3)	0.1505 (12)	0.2393 (7)	0.039*	
H11B	0.586 (3)	0.2484 (12)	0.1971 (8)	0.039*	
H11C	0.726 (3)	0.1670 (11)	0.2640 (7)	0.039*	

### Atomic displacement parameters (Å^2^)

**Table:**

	*U*^11^	*U*^22^	*U*^33^	*U*^12^	*U*^13^	*U*^23^
O1	0.0137 (5)	0.0268 (6)	0.0384 (6)	0.0012 (4)	0.0041 (4)	0.0058 (4)
N1	0.0109 (6)	0.0207 (7)	0.0258 (6)	−0.0004 (5)	0.0047 (5)	0.0015 (5)
C1	0.0123 (7)	0.0189 (8)	0.0213 (7)	0.0010 (6)	−0.0006 (5)	0.0026 (6)
C2	0.0144 (7)	0.0225 (8)	0.0200 (7)	0.0055 (6)	0.0009 (5)	0.0025 (6)
C3	0.0219 (7)	0.0218 (9)	0.0243 (7)	0.0049 (7)	0.0022 (6)	−0.0031 (6)
C4	0.0232 (8)	0.0203 (8)	0.0285 (8)	−0.0022 (7)	0.0024 (7)	0.0029 (7)
C5	0.0211 (7)	0.0272 (9)	0.0214 (7)	−0.0004 (7)	0.0048 (6)	0.0040 (6)
C6	0.0154 (7)	0.0230 (8)	0.0199 (7)	0.0016 (6)	−0.0001 (6)	0.0006 (6)
C7	0.0163 (7)	0.0220 (8)	0.0193 (7)	0.0017 (6)	0.0030 (5)	−0.0013 (6)
C8	0.0174 (7)	0.0227 (8)	0.0281 (8)	−0.0003 (7)	0.0051 (6)	0.0015 (6)
C9	0.0236 (8)	0.0234 (9)	0.0556 (11)	0.0020 (7)	0.0068 (8)	0.0068 (8)
C10	0.0226 (8)	0.0282 (9)	0.0243 (8)	0.0021 (7)	0.0072 (6)	−0.0002 (6)
C11	0.0270 (8)	0.0284 (9)	0.0224 (7)	−0.0009 (7)	0.0065 (6)	−0.0030 (6)

### Geometric parameters (Å, °)

**Table:**

O1—C7	1.2317 (15)	C6—C11	1.5073 (18)
N1—C7	1.3509 (16)	C7—C8	1.5101 (18)
N1—C1	1.4299 (16)	C8—C9	1.508 (2)
N1—H1N	0.889 (14)	C8—H8A	0.998 (14)
C1—C6	1.4000 (17)	C8—H8B	0.968 (13)
C1—C2	1.4012 (17)	C9—H9A	0.988 (16)
C2—C3	1.3928 (18)	C9—H9B	0.987 (15)
C2—C10	1.4994 (19)	C9—H9C	1.020 (17)
C3—C4	1.3828 (18)	C10—H10A	0.986 (15)
C3—H3	0.969 (13)	C10—H10B	0.982 (15)
C4—C5	1.3834 (19)	C10—H10C	0.981 (14)
C4—H4	0.957 (13)	C11—H11A	0.978 (15)
C5—C6	1.3952 (18)	C11—H11B	1.005 (14)
C5—H5	0.973 (13)	C11—H11C	1.003 (15)
			
C7—N1—C1	122.69 (11)	C9—C8—C7	113.27 (12)
C7—N1—H1N	116.6 (8)	C9—C8—H8A	110.7 (7)
C1—N1—H1N	118.8 (8)	C7—C8—H8A	105.7 (7)
C6—C1—C2	121.59 (12)	C9—C8—H8B	112.1 (8)
C6—C1—N1	120.03 (11)	C7—C8—H8B	109.7 (8)
C2—C1—N1	118.38 (11)	H8A—C8—H8B	104.9 (11)
C3—C2—C1	118.17 (12)	C8—C9—H9A	111.4 (9)
C3—C2—C10	120.71 (12)	C8—C9—H9B	112.7 (9)
C1—C2—C10	121.11 (12)	H9A—C9—H9B	107.5 (12)
C4—C3—C2	121.19 (13)	C8—C9—H9C	111.2 (8)
C4—C3—H3	119.6 (8)	H9A—C9—H9C	106.5 (13)
C2—C3—H3	119.2 (8)	H9B—C9—H9C	107.3 (12)
C3—C4—C5	119.76 (13)	C2—C10—H10A	112.7 (8)
C3—C4—H4	118.4 (8)	C2—C10—H10B	113.0 (8)
C5—C4—H4	121.8 (8)	H10A—C10—H10B	105.1 (12)
C4—C5—C6	121.20 (13)	C2—C10—H10C	110.5 (8)
C4—C5—H5	118.0 (7)	H10A—C10—H10C	107.2 (12)
C6—C5—H5	120.8 (7)	H10B—C10—H10C	108.0 (11)
C5—C6—C1	118.05 (12)	C6—C11—H11A	111.7 (9)
C5—C6—C11	119.78 (12)	C6—C11—H11B	110.3 (8)
C1—C6—C11	122.17 (12)	H11A—C11—H11B	112.9 (11)
O1—C7—N1	122.39 (13)	C6—C11—H11C	111.1 (8)
O1—C7—C8	121.57 (12)	H11A—C11—H11C	103.2 (11)
N1—C7—C8	116.02 (11)	H11B—C11—H11C	107.3 (11)
			
C7—N1—C1—C6	65.81 (15)	C4—C5—C6—C1	−0.93 (19)
C7—N1—C1—C2	−113.86 (13)	C4—C5—C6—C11	178.87 (12)
C6—C1—C2—C3	−1.77 (18)	C2—C1—C6—C5	2.20 (17)
N1—C1—C2—C3	177.88 (11)	N1—C1—C6—C5	−177.45 (11)
C6—C1—C2—C10	178.15 (12)	C2—C1—C6—C11	−177.59 (12)
N1—C1—C2—C10	−2.20 (17)	N1—C1—C6—C11	2.75 (17)
C1—C2—C3—C4	0.06 (18)	C1—N1—C7—O1	−7.20 (19)
C10—C2—C3—C4	−179.86 (12)	C1—N1—C7—C8	171.64 (11)
C2—C3—C4—C5	1.17 (19)	O1—C7—C8—C9	−27.34 (19)
C3—C4—C5—C6	−0.7 (2)	N1—C7—C8—C9	153.81 (13)

### Hydrogen-bond geometry (Å, °)

**Table:**

*D*—H···*A*	*D*—H	H···*A*	*D*···*A*	*D*—H···*A*
N1—H1N···O1^i^	0.889 (14)	2.065 (14)	2.9352 (15)	165.9 (12)

Symmetry codes: (i) *x*−1, *y*, *z*.
